# Cardiac-MRI Predicts Clinical Worsening and Mortality in Pulmonary Arterial Hypertension: A Systematic Review and Meta-Analysis

**DOI:** 10.1016/j.jcmg.2020.08.013

**Published:** 2021-05

**Authors:** Samer Alabed, Yousef Shahin, Pankaj Garg, Faisal Alandejani, Christopher S. Johns, Robert A. Lewis, Robin Condliffe, James M. Wild, David G. Kiely, Andrew J. Swift

**Affiliations:** a Department of Infection, Immunity and Cardiovascular Disease, University of Sheffield, Sheffield, United Kingdom; b Department of Clinical Radiology, Sheffield Teaching Hospitals, Sheffield, United Kingdom; c Sheffield Pulmonary Vascular Disease Unit, Royal Hallamshire Hospital, Sheffield, United Kingdom; d INSIGNEO, Institute for In Silico Medicine, University of Sheffield, United Kingdom

**Keywords:** cardiac MRI, CMR, meta-analysis, mortality, PAH, pulmonary arterial hypertension, prognosis, systematic review, CMR, cardiac magnetic resonance, CTD, connective tissue disease, IPAH, idiopathic pulmonary arterial hypertension, LV, left ventricular, LVEDVI, left ventricular end-diastolic volume index, mPAP, mean pulmonary artery pressure, PAH, pulmonary arterial hypertension, PH, pulmonary hypertension, RV, right ventricular, RVEDVI, right ventricular end-diastolic volume index, RVEF, right ventricular ejection fraction, RVESVI, right ventricular end-systolic volume index, RVMI, right ventricular mass index, VMI, ventricular mass index

## Abstract

**Objectives:**

This meta-analysis evaluates assessment of pulmonary arterial hypertension (PAH), with a focus on clinical worsening and mortality.

**Background:**

Cardiac magnetic resonance (CMR) has prognostic value in the assessment of patients with PAH. However, there are limited data on the prediction of clinical worsening, an important composite endpoint used in PAH therapy trials.

**Methods:**

The Cochrane Central Register of Controlled Trials, MEDLINE, EMBASE, and Web of Science databases were searched in May 2020. All CMR studies assessing clinical worsening and the prognosis of patients with PAH were included. Pooled hazard ratios of univariate regression analyses for CMR measurements, for prediction of clinical worsening and mortality, were calculated.

**Results:**

Twenty-two studies with 1,938 participants were included in the meta-analysis. There were 18 clinical worsening events and 8 deaths per 100 patient-years. The pooled hazard ratios show that every 1% decrease in right ventricular (RV) ejection fraction is associated with a 4.9% increase in the risk of clinical worsening over 22 months of follow-up and a 2.1% increase in the risk of death over 54 months. For every 1 ml/m^2^ increase in RV end-systolic volume index or RV end-diastolic volume index, the risk of clinical worsening increases by 1.3% and 1%, respectively, and the risk of mortality increases by 0.9% and 0.6%. Every 1 ml/m^2^ decrease in left ventricular stroke volume index or left ventricular end-diastolic volume index increased the risk of death by 2.5% and 1.8%. Left ventricular parameters were not associated with clinical worsening.

**Conclusions:**

This review confirms CMR as a powerful prognostic marker in PAH in a large cohort of patients. In addition to confirming previous observations that RV function and RV and left ventricular volumes predict mortality, RV function and volumes also predict clinical worsening. This study provides a strong rationale for considering CMR as a clinically relevant endpoint for trials of PAH therapies.

Pulmonary arterial hypertension (PAH) is characterized by remodeling of the distal pulmonary arteries, leading to an increase in pulmonary vasculature resistance, reduced compliance, and elevated pulmonary artery pressure (1, 2, 3). Untreated, PAH has high morbidity and mortality that are closely linked to right ventricular (RV) dysfunction (2). A new diagnosis of PAH increases the risk of death at 1 year fivefold (4). However, over the last 20 years, treatment advancements have led to an increase in median survival from 3 to 7 years (4, 5, 6), although development of new therapies is needed.

Recently, clinical studies of PAH therapies have moved from assessing exercise capacity and pulmonary hemodynamics to using composite endpoints. One such approach uses the time to clinical worsening. Clinical worsening events include hospitalization, disease progression, and unsatisfactory long-term clinical response, in addition to mortality (7). However, given the large number of patients required and the expense of conducting such event-driven studies, cardiac magnetic resonance (CMR) has recently been explored as a primary endpoint to evaluate PAH therapies (8).

CMR is the gold standard method of measuring RV function, volumes, and mass, and it is an established prognostic and therapy response tool (1,9,10). In 2015, a meta-analysis assessed the prognostic value of CMR measurements in 5 studies with 332 participants (9); however, no data were reported on clinical worsening. Since then, multiple new studies assessing clinical worsening in addition to mortality have been published in PAH.

The current meta-analysis also includes unpublished supplemental data on CMR metrics from 16 previously published studies, which allowed us to provide new data on the utility of CMR to predict clinical worsening in addition to mortality. The goal of the current study therefore was to review the evidence for CMR metrics to predict clinical worsening and mortality in patients with PAH.

## Methods

The review was prospectively registered with The International Prospective Register of Systematic Reviews on December 12, 2019 (ID: CRD42019160296). The Preferred Reporting Items for Systematic reviews and Meta-Analysis guidelines were followed (11). Ethical approval was not required for this meta-analysis because it was based on published literature and did not recruit patients.

### Criteria for considering studies for review

Studies of all forms of PAH (including idiopathic PAH [IPAH], heritable, drug- and toxin-induced PAH, and PAH associated with connective tissue disease [CTD]; congenital heart disease; HIV infection; and portal hypertension) were considered for inclusion in the meta-analysis. For studies including patients with different forms of pulmonary hypertension (PH), data from these studies were incorporated only if the PAH cohort was separately described or the PAH participants formed at least one-half of the study population. To obtain additional data on patients with PAH, which may have been collected but not published, authors were contacted and supplemental data requested. Case reports or small cases series of <10 participants were excluded. Data collected included any clinically relevant outcomes such as hospitalization due to heart failure, disease progression, unsatisfactory long-term clinical response, and death. To allow for analysis, only studies that reported Cox regression hazard ratios expressed per unit of measurement were included. One study reporting dichotomized hazard ratios was excluded because raw hazard ratios could not be obtained after contacting the study author.

### Search methods for identification of studies

The following databases were systematically searched for relevant studies on May 13, 2020: Cochrane Central Register of Controlled Trials (Central) (Issue 1, May 2020), MEDLINE (ProQuest, 1946 to May 6, 2020), EMBASE (Ovid, 1974 to 2020 Week 20), and Web of Science (to May 13, 2020). The search strategy used is outlined in Supplemental Appendix 1. The reference lists of all relevant articles identified during the full-text screening were scrutinized for relevant studies.

### Data collection and analysis

Details about the selection of studies, data extraction and management, and statistical analysis and data synthesis are provided in Supplemental Appendix 2.

## Results

### Search results

Our comprehensive search identified a total of 22 studies that were included in the meta-analysis (12, 13, 14, 15, 16, 17, 18, 19, 20, 21, 22, 23, 24, 25, 26, 27, 28, 29, 30, 31, 32, 33). The details of the literature search are presented in the supplemental materials, including a Preferred Reporting Items for Systematic Reviews and Meta-Analysis flow diagram (Supplemental Appendix 3).

### Description of included studies

#### Study design

The review includes 14 case series and 8 case-control studies. Prospective recruitment was performed in 12 studies, and consecutive inclusion was reported in 10 studies. Apart from Leng et al. (21), all studies were single tertiary center studies. The studies were published between 2007 and 2020, with 16 studies including 1,606 participants published since the previous meta-analysis in 2015. Most studies (18 studies) had a small sample size of <100 patients, with the largest study by Swift et al. (26) including 576 participants.

### Population

The 22 studies were conducted in 10 different countries and included 2,149 participants. A total of 1,938 participants were included in the meta-analysis, of whom 97% had PAH and 3% had other types of PH. IPAH comprised 51% and CTD-PAH 26% of the PAH population. Dawes et al. (31), de Siqueira et al. (16), and Jose et al. (30) kindly provided additional data that allowed identification of patients with PAH from a mixed PH cohort.

**Figure 1:**
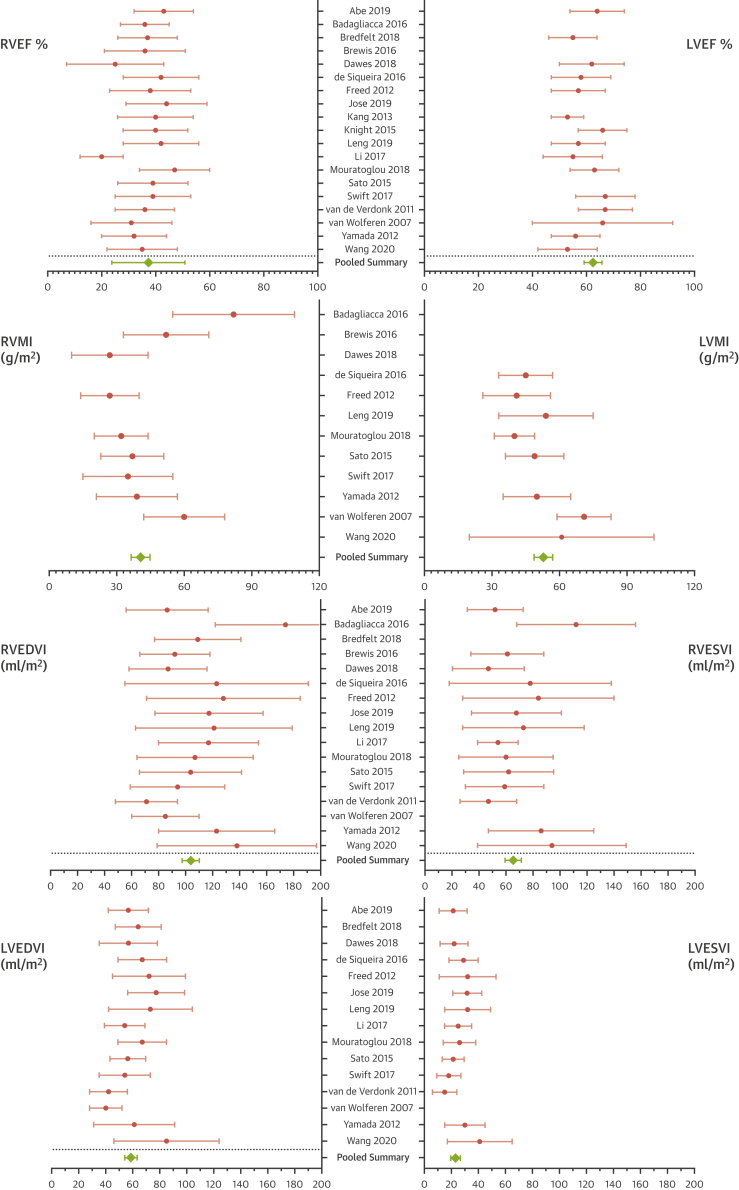
Pooled Baseline CMR Characteristics The included studies had homogeneous mean baseline cardiac magnetic resonance (CMR) measurements as shown by the overlapping confidence intervals, with relatively more heterogeneity in right ventricular mass and volumes. The overall pooled mean CMR measurements show moderately impaired right ventricular function and volumes at baseline and indicate a relatively advanced disease. LVEDVI = left ventricular end-diastolic volume index; LVEF = left ventricular ejection fraction; LVESVI = left ventricular end-systolic volume index; LVMI = left ventricular mass index; RVEDVI = right ventricular end-diastolic volume index; RVEF = right ventricular ejection fraction; RVMI = right ventricular mass index; RVESVI = right ventricular end-systolic volume index.

Participants were age 52 ± 15 years, with a female predominance (68%) and a pooled average mean pulmonary artery pressure (mPAP) of 49 ± 15 mm Hg and RV ejection fraction (RVEF) of 37 ± 14%. Details of the included studies are presented in Table 1. The pooled baseline CMR measurements are shown in Figure 1.

**Table 1:** Characteristics of Included Studies

First Author **(Ref. #)**, Year	Country	Design	Study Period	Size	Female (%)	Age, yrs	**mPAP (mm Hg)**	IPAH	CTD	CHD	Other PAH	Other PH	Follow-up (months)	Death	Clinical Events
Abe et al. (12), 2019	Japan	CC	2008–2018	65	88	56 ± 15	34 ± 11		54		11		42 (13–86)	9 (14)	
Badagliacca et al. (13), 2016	Italy	PCS	2011–2013	74	59	55 ± 13	48 ± 13	74					18 (2–33)		31 (42)
Bredfelt et al. (14), 2018	Sweden	RCS	2003–2015	75	71	57 ± 19	45 ± 11	33	33			9	28	29 (39)	7 (9)
Brewis et al. (15), 2016	U.K.	CC	2004–2014	140	66	55 ± 16	48 ± 13	75	53	1	11		69	61 (44)	
Dawes et al. (31), 2018∗	U.K.	CC	2004–2013	256	44	65 ± 17	43 ± 16	57			31	168	48 (24–68)	34 (39)	
de Siqueira et al. (16), 2016∗	U.S.	CC	2003–2013	93	74	52 ± 12	40 ± 15	23	25		22	23	24 (6–52)		25 (36)
Freed et al. (12), 2012	U.S.	PCS	2009–2010	58	74	53 ± 14	49 ± 16	24			20	14	10 ± 6	6 (10)	13 (22)
Gan et al. (18), 2007	Holland	CC	2001–2005	70	79	50 ± 15	53 ±14	49	16		5		48	18 (26)	
Grapsa et al. (32), 2020	U.K.	PCS	NR	30	80	47 ± 5	NR	30					24 (17–24)	8 (26)	
Jose et al. (30), 2020∗	U.S.	RCS	2013–2019	38	68	51 ± 17	45 ± 15	18				20	20 (11–35)		4 (11)
Kang et al. (19), 2013	South Korea	PCS	2009–2010	30	74	45 ± 13	51 ± 23	19	2	7	2		17 ± 7	1 (3)	6 (20)
Knight et al. (20), 2015	U.K.	CC	2012–2013	40	75	50 ± 5	46 ± 13	12	20			8	20 ± 8	1 (3)	8 (20)
Leng et al. (21), 2019	Singapore	CC	2015–2018	80	79	37 ± 15	56 ± 22	21	10	40	9		24 (2–57)	6 (8)	8 (10)
Li et al. (22), 2017	China	PCS	2010–2013	41	71	29 ± 9	61 ± 16	41					27 (21–41)	7 (17)	10 (24)
Mouratoglou et al. (23), 2018	Greece	PCS	NR	36	78	51 ± 14	NR	12	7	9	2	6	20 (4-37)	0	14 (39)
Sato et al. (24), 2015	Japan	PCS	2009–2013	68	76	55 ± 22	37 ± 11	10	17		4	37	24 (9-34)	10 (15)	6 (9)
Simpson et al. (25), 2019	U.S.	PCS	2007–2014	64	91	57 ± 11	NR	22	42				50 (29–66)	30 (46)	
Swift et al. (26), 2017	U.K.	RCS	2008–2015	576	54	57 ± 16	48 ± 13	260	195	63	58		42 (17–142)	221 (38)	
van de Veerdonk et al. (27), 2011	Holland	PCS	2002–2007	110	76	53 ± 15	49 ± 16	73	20		17		59 (30–74)	30 (27)	2 (2)
van Wolferen et al. (28), 2007	Holland	PCS	1999–2005	64	73	43 ± 13	56 ± 13	64					32 ± 16	19 (30)	
Wang et al. (33), 2020	China	CC	2013–2018	100	70	37 ± 14	62 ± 22	33	8	58	1		15 (7–27)	9 (9)	21 (21)
Yamada et al. (29), 2012	Japan	RCS	2003–2010	41	71	39 ± 14	51 ± 14	41					44 ± 25		32 (78)

Values are median (range), n (%), mean ± SD, or n, unless otherwise indicated.

CC = case-control; CHD = congenital heart disease; CTD = connective tissue disease; IPAH = idiopathic pulmonary arterial hypertension; mPAP = mean pulmonary artery pressure; NR = not reported; PCS = prospective case series; PAH = pulmonary arterial hypertension; PH = pulmonary hypertension; RCS = retrospective case series; U.K. = United Kingdom; U.S. = United States.

∗ Only patients with pulmonary arterial hypertension (PAH) were included in the analysis.

#### Methodological quality of included studies

One-half of the studies had a prospective design, consecutive recruitment of participants, and reported blinding of CMR readers to patient clinical data. The main concern for bias is the small sample size of <100 participants in 18 of the 22 included studies. All studies were performed at PH referral centers and are therefore at risk for referral bias. The detailed results of the quality assessment are described in Supplemental Appendix 4, including a QUIPS (Quality in Prognostic Studies) risk of bias figure.

#### Meta-analyses of CMR indices

**Figure 2:**
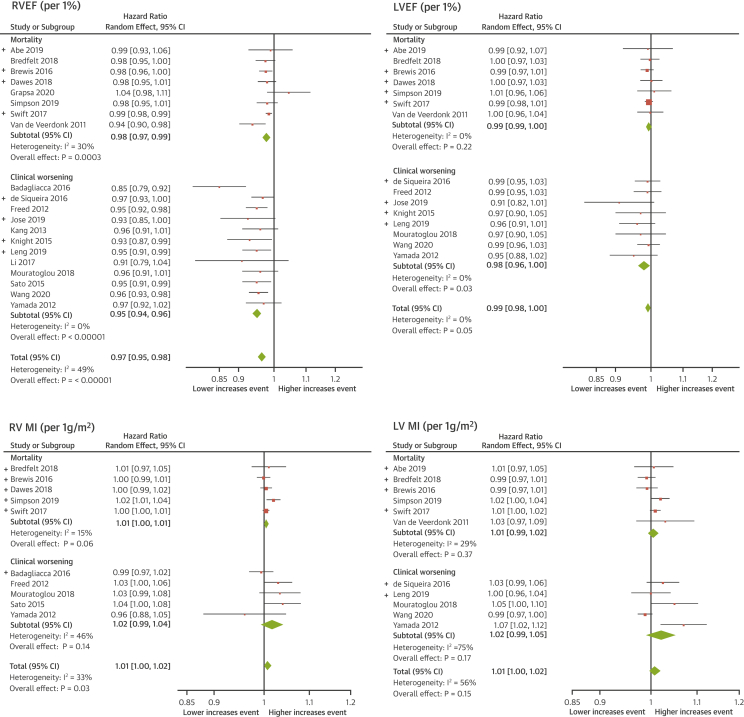
Meta-Analyses of RV and LV Function and Mass The meta-analyses of right ventricular (RV) and left ventricular (LV) function and mass showed that RVEF and RVMI are significant prognostic markers. RVEF could predict clinical worsening separate from mortality, whereas RVMI is a nonspecific prognostic marker. Unpublished data are indicated by (+). CI = confidence interval; other abbreviations as in Figure 1.

**Figure 3:**
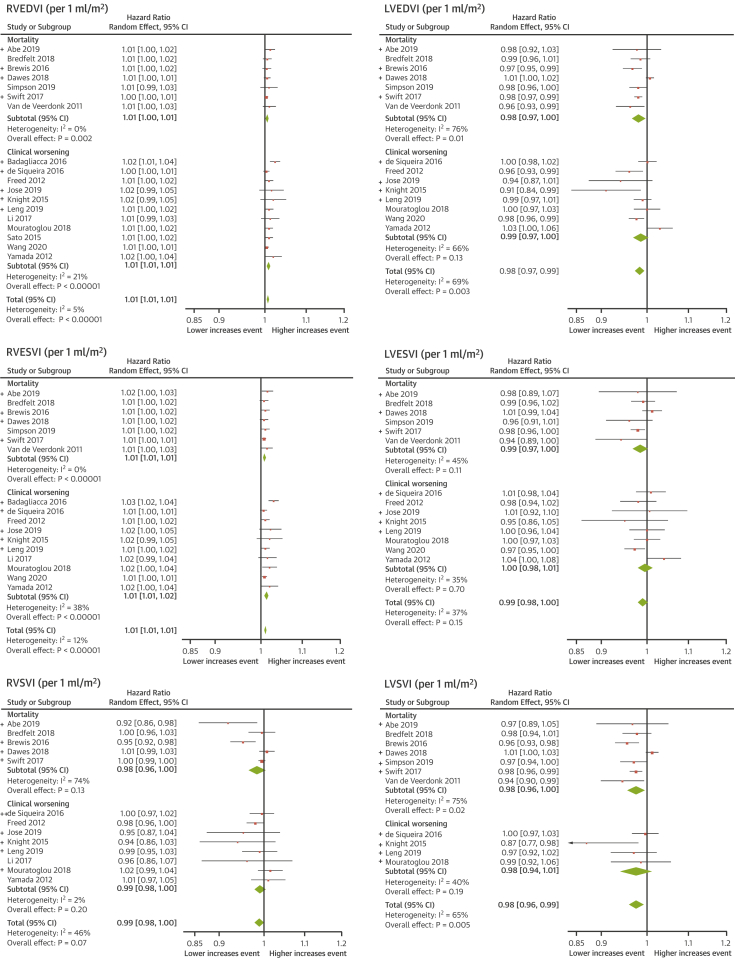
Meta-Analyses of RV And LV Volume Measurements RV and LV volumes are significant prognostic markers. A decrease in RV volumes can predict mortality and clinical worsening, whereas an increase in LV volumes indicates an increased risk for death only. Unpublished data are indicated by (+). LVSVI = left ventricular stroke volume index; RVSVI = right ventricular stroke volume index; other abbreviations as in Figures 1 and 2.

Clinical worsening was analyzed separately to mortality in a subgroup analysis. In 10 studies, providing data exclusively on mortality, 459 deaths (36%) in 1,282 participants occurred over a mean follow-up of 54 ± 5 months (8 deaths per 100 patient-years). The hazard ratios of the meta-analysis are presented in Table 2. A drop of 1% in RVEF increased the risk of death by 2.1%. A decrease of 1 ml/m^2^ in left ventricular (LV) stroke volume index or LV end-diastolic volume index (LVEDVI) increased the risk of death by 2.5% and 1.8%, respectively. An increase in RV volumes, right ventricular end-systolic volume index (RVESVI) or right ventricular end-diastolic volume index (RVEDVI), by 1 ml/m^2^ increased the risk of mortality by 0.9% and 0.6%. The forest plots for RV and LV function and mass are shown in Figure 2 and forests plots for RV and LV volume measurements in Figure 3.

**Table 2:** Results of Meta-Analyses of Univariate Hazard Ratios for CMR Measurements

CMR Measurement	Overall Meta-Analysis		Mortality Outcome		Clinical Worsening	
	HR (95% CI)	Studies (n)	HR (95% CI)	Studies (n)	HR (95% CI)	Studies (n)
RVEF	0.965 (0.954–0.976)	20 (1,804)	0.979 (0.969–0.990)	8 (1,148)	0.951 (0.939–0.964)	12 (656)
RVEDVI	1.007 (1.005–1.010)	18 (1,744)	1.006 (1.003–1.008)	7 (1,118)	1.010 (1.006–1.013)	11 (626)
RVESVI	1.010 (1.008–1.013)	17 (1,676)	1.009 (1.005–1.012)	7 (1,118)	1.013 (1.008–1.018)	10 (558)
RVSVI	0.989 (0.978–1.001)	13 (1,328)	0.984 (0.965–1.004)	5 (944)	0.992 (0.979–1.004)	8 (384)
LVEF	0.992 (0.984–1.000)	15 (1,561)	0.994 (0.986–1.003)	7 (1,118)	0.980 (0.963–0.998)	8 (443)
LVEDVI	0.985 (0.974–0.995)	15 (1,561)	0.982 (0.968–0.996)	7 (1,118)	0.986 (0.969–1.004)	8 (443)
LVESVI	0.991 (0.979–1.003)	14 (1,421)	0.985 (0.967–1.003)	6 (978)	0.997 (0.979–1.014)	8 (443)
LVSVI	0.976 (0.960–0.993)	11 (1,344)	0.975 (0.956–0.995)	7 (1,118)	0.976 (0.940–1.012)	4 (226)
RVMI	1.008 (1.001–1.016)	10 (1,220)	1.006 (1.000–1.012)	5 (943)	1.018 (0.994–1.041)	5 (277)
LVMI	1.009 (0.997–1.020)	11 (1,357)	1.005 (0.995–1.016)	6 (1,030)	1.022 (0.991–1.053)	5 (327)

CI = confidence interval; CMR = cardiac magnetic resonance; HR = hazard ratio; LVEDVI = left ventricular end-diastolic volume index; LVEF = left ventricular ejection fraction; LVESVI = left ventricular end-systolic volume index; LVMI = left ventricular mass index; LVSVI = left ventricular stroke volume index; RVEDVI = right ventricular end-diastolic volume index; RVEF = right ventricular ejection fraction; RVMI = right ventricular mass index; RVESVI = right ventricular end-systolic volume index; RVSVI = right ventricular stroke volume index.

In 12 studies, providing data on clinical worsening, 218 (33%) events occurred in 656 participants over a mean follow-up of 22 ± 4 months (18 clinical worsening events per 100 patient-years). The composite outcome of clinical worsening included hospitalization for heart failure (42%), escalation to prostacyclin treatment (18%), deterioration in World Health Organization functional class (3%), a reduction in exercise capacity (2%), need for lung transplantation (2%), nonspecified aforementioned nonfatal event (14%), and all-cause death (19%). RV but not LV volumetric and functional measurements predicted clinical worsening. A 1% deterioration in RVEF was associated with a 4.9% increase in the risk of clinical worsening, and a 1 ml/m^2^ increase in RVESVI or RVEDVI was associated with an increase of clinical worsening of 1.3% and 1%, respectively.

### Heterogeneity

There was high statistical heterogeneity in the overall result of LV mass index, LVSVI and LVEDVI, and moderate heterogeneity in RVEF, LV end-systolic volume index, and RVSVI. A meta-regression model of the logHR of these variables showed no evidence of a linear relationship with age, male sex, 6-min walking test, or right heart catheterization parameters (Supplemental Appendix 5). There may, however, be sources of heterogeneity that could not be assessed in a meta-regression analysis where not enough data were available. There were differences in the types of clinical worsening events used as endpoints, length of follow-up, and the subgroups of PAH studied. Other causes of heterogeneity may include variation in baseline CMR measurements (Figure 1), disease severity, and treatment status. There is also geographic variation: 11 studies were from European centers, 4 from the United States, and 7 from Japan, South Korea, Singapore, and China.

### Publication Bias

Unpublished data from previously published studies were obtained to reduce the risk of publication bias (Supplemental Appendix 6). The results of Van Wolferen 2007 (28) had very large effect sizes and standard errors, following discussion with the co-authors we understand this is due to scaling of the CMR measurements to the standard deviation rather than the unit of measurement. We therefore decided not to pool the results of Van Wolferen 2007 with the rest of the studies due to the different unit of scaling used. The funnel plots of RVEF, LVEF, RVMI and LVEDVI showed minor asymmetry which could indicate that a small study with with extreme effect sizes was not published (Supplemental Appendix 7).

## Discussion

To the best of our knowledge, this paper is the largest meta-analysis of CMR imaging in patients with PAH and the first to report on clinical worsening in addition to mortality. We have confirmed that CMR imaging is a powerful prognostic marker in a large cohort of patients from multiple institutions, across several continents and using different imaging platforms. In addition, we have shown that CMR imaging predicts clinical worsening in patients with PAH. Our findings highlight the clinical utility of CMR imaging and support further evaluation of this modality as a clinically meaningful trial endpoint for the assessment of new therapies for PAH (Central Illustration, Table 3).

**Central Illustration:**
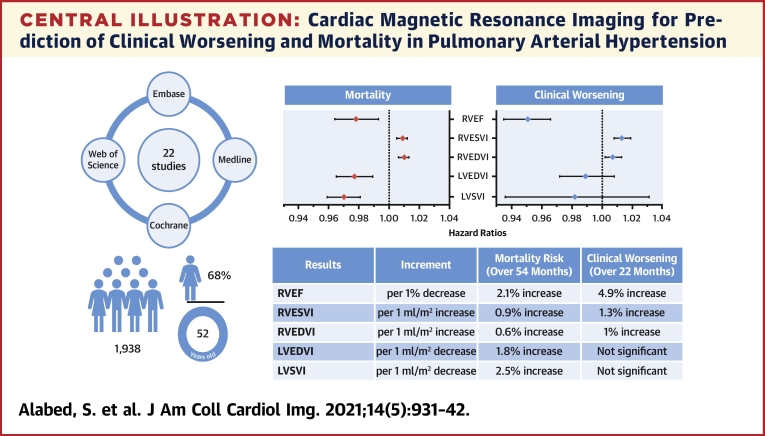
Cardiac Magnetic Resonance Imaging for Prediction of Clinical Worsening and Mortality in Pulmonary Arterial Hypertension Pooled results for mortality and clinical worsening are presented in the forest plots and described in the table underneath for various factors. The literature search details and demographic characteristics of the meta-analysis cohort are shown on the left. LVEDVI = left ventricular end-diastolic volume index; LVESVI = left ventricular end-systolic volume index; LVSVI = left ventricular stroke volume index; RVEDVI = right ventricular end-diastolic volume index; RVEF = right ventricular ejection fraction; RVESVI = right ventricular end-systolic volume index.

**Table 3:** Summary of Findings

Review question	What are the CMR predictors for clinical worsening and mortality in patients with PAH?		
Population	1,938 participants, including 68% female subjects, aged 52 ± 15 yrs. Participants had more advanced disease and intermediate to high risk for 1-yr mortality		
Follow-up	22 ± 4 months for clinical worsening and 54 ± 5 months for mortality		
Setting	Tertiary pulmonary hypertension referral centers		
Studies	Case series and case-control studies		
Quality of evidence	Some concerns for bias due to small sample sizes, retrospective design, lack of blinding in most studies and non-consecutive inclusion in half of the studies.		
**Results**	**Increment**	**Clinical Worsening (Over 22 Months)**	**Mortality Risk (Over 54 Months)**
RVEF	per 1% decrease	4.9% increase	2.1% increase
RVESVI	per 1 ml/m^2^ increase	1.3% increase	0.9% increase
RVEDVI	per 1 ml/m^2^ increase	1% increase	0.6% increase
LVEDVI	per 1 ml/m^2^ decrease	Not significant	1.8% increase
LVSVI	per 1 ml/m^2^ decrease	Not significant	2.5% increase

Abbreviations as in Tables 1 and 2.

Clinical worsening as a composite endpoint has been shown to predict mortality (34) and has established itself as a primary efficacy endpoint in trials of PAH therapies (7,35). Although heart failure and all-cause mortality are included in all PAH trials using a composite clinical worsening endpoint, these trials vary in their inclusion and definition of progression markers, such as change in exercise tolerance or functional capacity (36), and they may use different thresholds to define a meaningful change (37). Nonetheless, study designs using time to clinical worsening have been increasingly adopted to evaluate PAH therapies. However, such studies require large numbers of participants and a prolonged period of follow-up, usually lasting for several years. As a consequence and given recent events such as the coronavirus disease-2019 pandemic, there has been a focus on considering clinical trial endpoints, which allow the impact of candidate therapies to be assessed over a shorter period and using an endpoint that correlates with clinically meaningful events.

In this meta-analysis, we have shown for the first time in a large cohort of patients that CMR-derived RV volumetric and functional metrics but not LV measurements predict clinical worsening. This information should be helpful to regulatory authorities who are keen to ensure that proposed trial endpoints have clinical relevance. In addition, this meta-analysis confirms the prognostic value of CMR metrics in a substantial cohort of patients, which has allowed an assessment of the impact of change on specific metrics concerning clinical worsening, including mortality. A 1% decrease in RVEF is associated with a 4.9% increase in the risk of clinical worsening and a 2.1% increase in the risk of death. In addition, a 1 ml/m^2^ increase in RV volumes is associated with a 0.6% to 0.9% increase in the risk of mortality and a 1% to 1.3% increase in the risk of clinical worsening. Although this incremental change in RV volumes is smaller than the 1.8% associated with a decrease in LV end-diastolic volume, the overall risk of mortality is more linked to RV volume, previously highlighted in large cohort studies (26,38). Specifically, the increase in RV volume due to dilation in response to an increase in afterload is substantially larger than the change in LV volume, occurring as a consequence of ventricular interaction (39), particularly in advanced disease in PAH when uncoupling of the right ventricle and its load occurs (40).

This meta-analysis has shown that an increased RV mass has prognostic value but does not predict clinical worsening. In PAH, an increase in RV mass and RV hypertrophy is likely to represent an appropriate adaptive response to an increase in afterload (41). In a CMR study in which patients were monitored over 5 years, RV wall thickness was not associated with increased mortality in patients who were judged to be clinically stable (39). Moreover, a disproportionate increase in right ventricular mass index (RVMI) compared with RVEDV indicates concentric hypertrophy and is associated with a favorable outcome in IPAH (13). Eccentric hypertrophy with a disproportionate increase in RVEDV compared with RVMI is considered a maladaptive response to increased afterload and is associated with a poor outcome (13,41). In IPAH, therefore, caution should be exercised when using mass measurements in isolation because they give incomplete information on RV adaptation. Further study of the relationship between RV mass and volume would be helpful. In CTD-PAH, in which the natural history of the disease is different, RVMI and ventricular mass index (VMI) seem to have greater prognostic value than RV function or volumes (17,30,31,42). A 10% increase in RVMI and VMI was associated with an increased risk of death of 10% to 15% (25). A VMI ≥0.7 was associated with 35% mortality at 1 year and 67% mortality at 2 years (42). RV hypertrophy in CTD-PAH may be an early prognostic marker for mortality rather than just an adaptive response to PH (25). This finding emphasizes the importance of considering the clinical context when using tools to assess prognosis.

Several additional CMR measurements have been shown in small studies to have prognostic value, and additional data are provided in Supplemental Appendix 8. These analyses include right atrial volume and area, pulmonary artery relative area change and distensibility, and the ratio of stroke volume/RV end-systolic volume. Additional CMR prognostic markers that were not assessed in the meta-analysis include myocardial strain analysis, myocardial T_1_ mapping, and late gadolinium enhancement. The small number of studies reporting these markers, or the absence of Cox regression analysis, prevented a meaningful pooling of their results. Strain analysis using feature tracking seems to be a promising prognostic marker (16,21,43); however, it needs to be evaluated further in a more extensive survival study. Late gadolinium enhancement (17,44,45) and T_1_ mapping (46) have an unclear additive prognostic value in PAH.

The meta-analysis is based on a population likely to have disease at the more severe end of the spectrum. The results may therefore not be generalizable to patients with more modest disease, in which age and comorbidity may have more of an impact on prognosis. A recently published, large well-designed study has shown that CMR could be used to establish thresholds for mortality risk in PAH (38). This study showed that CMR metrics can be used to improve risk stratification when incorporated into the French Registry approach or REVEAL (Registry to Evaluate Early and Long-Term Pulmonary Arterial Hypertension Disease Management) risk scores (47,48). In the study by Lewis et al. (38), an RVEF <37%, an RVESVI of >54 ml/m^2^, and LVEDVI of <52 ml/m^2^ were associated with a high risk of mortality. In this meta-analysis, the pooled RVEF was 37%, RVESVI 63 ml/m^2^, and LVEDVI 57 ml/m^2^. All included studies used the current guideline criteria of an mPAP threshold ≥25 mm Hg. A new threshold of >20 mm Hg with a pulmonary vascular resistance ≥3 Wood units has recently been proposed as a hemodynamic definition for PAH, being 2 SDs above the normal threshold (49). There remains a lack of evidence therefore regarding the prognostic value of CMR in patients who have modest PAH and those with mPAP >20 mm Hg, in whom other factors may be more of a driver to clinical worsening and mortality.

### Study limitations

An extensive systematic literature search was performed, and a pre-published protocol was followed. However, a potential limitation of this study is that inclusion was assessed by a single investigator. Any doubt regarding study selection was discussed with another investigator, however. This meta-analysis contains previously unpublished data for participants included in previously published studies. However, this approach has allowed improved data completeness and additional analysis. The included studies including supplemental data are indicated by (+) in Figures 2 and 3. The unpublished univariate hazard ratios are provided in Supplemental Appendix 5. Patients in this study included a cohort with a predominantly intermediate and high risk of 1-year mortality and likely represent a cohort with more severe disease. Although the results of the meta-analysis suggest that CMR imaging, as performed in expert centers, strongly associates with outcomes, some caution is warranted in its application in less-experienced centers given the limited existence of multicenter studies. In some instances, heterogeneity is high, and greater caution in interpretation is therefore indicated. Finally, only limited data are provided on the potential of CMR metrics such as myocardial strain analysis, right atrial size, pulmonary artery wall stiffness, and four-dimensional flow parameters and the application of artificial intelligence approaches to large imaging datasets, which may add clinical value.

## Conclusions

This meta-analysis is the first to study the role of CMR in the prediction of clinical worsening in PAH. We have shown that CMR can predict clinical worsening, in addition to confirming its prognostic role, in a large cohort of patients with PAH. This study provides a strong rationale for considering CMR as a clinical trial endpoint.

Perspectives **COMPETENCY IN MEDICAL KNOWLEDGE:** Clinical worsening is an important composite endpoint used in therapy trials in PAH. In a meta-analysis of 1,938 participants with PAH, we showed that CMR predicts clinical worsening and has prognostic value. In a meta-regression, we have also shown that CMR predicts clinical worsening and mortality independent of age, sex, pulmonary hemodynamics, and walking distance. This study provides further data supporting the clinical utility of CMR in patients with PAH.

**TRANSLATIONAL OUTLOOK:** The findings of this meta-analysis provide a strong rationale for future research to consider CMR as a clinically relevant endpoint for therapy trials in PAH.

## Funding Support and Author Disclosures

The study was supported by the Wellcome Trust grants 215799/Z/19/Z and 205188/Z/16/Z. The funder did not have any role in the design and conduct of the study; in the collection, analysis, and interpretation of the data; or in the preparation, review, and approval of the paper. The authors have reported that they have no relationships relevant to the contents of this paper to disclose.
