# Supplementary material for: Cardiac-MRI Predicts Clinical Worsening and Mortality in Pulmonary Arterial Hypertension: A Systematic Review and Meta-Analysis
Source: JACC Cardiovasc Imaging. 2021 May;14(5):931–42. doi: 10.1016/j.jcmg.2020.08.013 (PMC7525356; doi:10.1016/j.jcmg.2020.08.013)
Supplement: Supplemental Appendix 1 to 7 [file mmc1.docx]

# Appendix 1: Search Strategy - 13/05/2020

## Medline

| 1 | exp "HYPERTENSION, PULMONARY"/ | (35,698) |
| --- | --- | --- |
| 2 | exp "PULMONARY HEART DISEASE"/ | (6,182) |
| 3 | (pulmonary vascular disease).ti,ab | (12,146) |
| 4 | (pulmonary ADJ2 hypertensi*).ti | (24,725) |
| 5 | (1 OR 2 OR 3 OR 4) | (51,604) |
| 6 | exp "MAGNETIC RESONANCE IMAGING"/ | (445,793) |
| 7 | (MRI* OR CMR*).ti,ab | (247,170) |
| 8 | (MR ADJ3 (imag* OR scan*)).ti,ab | (65,074) |
| 9 | (6 OR 7 OR 8) | (542,352) |
| 10 | (prognos* OR predict* OR clinical* OR outcome* OR associa* OR risk* OR death OR mortal* OR surviv* OR follow-up OR course OR progress* OR deteriorat*).ti,ab | (11,028,927) |
| 11 | exp MORTALITY/ | (376,208) |
| 12 | exp PROGNOSIS/ | (1,639,042) |
| 13 | (10 OR 11 OR 12) | (11,469,053) |
| 14 | (5 AND 9 AND 13) | ( 809) |

## Embase

| 1 | exp "PULMONARY HYPERTENSION"/ | 90,150 |
| --- | --- | --- |
| 2 | exp "PULMONARY VASCULAR DISEASE"/ | 5,045 |
| 3 | exp "PULMONARY HEART DISEASE"/ | 101,130 |
| 4 | (pulmonary ADJ2 hypertensi*).ti,ab | 37,760 |
| 5 | (1 OR 2 OR 3 OR 4) | 102,005 |
| 6 | exp "CARDIOVASCULAR MAGNETIC RESONANCE"/ | 35,176 |
| 7 | exp MAGNETIC RESONANCE IMAGING/ | 947,762 |
| 8 | exp CARDIAC IMAGING/ | 10,836 |
| 9 | (MRI* OR CMR*).ti,ab | 435,744 |
| 10 | (MR ADJ3 (imag* OR scan*)).ti,ab | 87,111 |
| 11 | (6 OR 7 OR 8 OR 9 OR 10) | 1,007,290 |
| 12 | (prognos* OR predict* OR clinical* OR outcome* OR associa* OR risk* OR death OR mortal* OR surviv* OR follow-up OR course OR progress* OR deteriorat*).ti,ab | 14,660,238 |
| 13 | exp MORTALITY/ | 1,058,168 |
| 14 | exp PROGNOSIS/ | 712,355 |
| 15 | (12 OR 13 OR 14) | 14,898,883 |
| 16 | (5 AND 12 AND 15) | 4,507 |

##

## Web of Science

| 1 | TS=((pulmon*) AND (hypertension OR vascular OR heart OR cardiac)) OR TS= (PULMONARY VASCULAR DISEASE) OR TS= (PULMONARY HYPERTENSION) | 158,583 |
| --- | --- | --- |
| 2 | TS=(CARDIOVASCULAR MAGNETSC RESONANCE) OR TS=(MAGNETSC RESONANCE IMAGING) OR TS=(CARDIAC IMAGING) OR TS=(MRI*) OR TS=(CMR*) OR TS=(MR NEAR (imag* OR scan*)) | 431,614 |
| 3 | TS=(prognos* OR predict* OR clinical* OR outcome* OR associa* OR risk* OR death OR mortal* OR surviv* OR follow-up OR course OR progress* OR deteriorat*) | 15,615,269 |
| 4 | #3 AND #2 AND #1 | 5,533 |

## Cochrane Central Library

| #1 | MeSH descriptor: [Hypertension, Pulmonary] explode all trees | 1035 |
| --- | --- | --- |
| #2 | MeSH descriptor: [Pulmonary Heart Disease] explode all trees | 66 |
| #3 | (pulmonary vascular disease):ti,ab,kw | 1485 |
| #4 | (pulmonary NEAR hypertensi*):ti,ab,kw (Word variations have been searched) | 3710 |
| #5 | #1 OR #2 OR #3 OR #4 | 4725 |
| #6 | MeSH descriptor: [Magnetic Resonance Imaging] explode all trees | 8002 |
| #7 | (MRI OR MRIs):ti,ab,kw (Word variations have been searched) | 23088 |
| #8 | ((MR NEAR (imag* OR scan*))):ti,ab,kw (Word variations have been searched) | 2221 |
| #9 | #6 OR #7 OR #8 | 27686 |
| #10 | (prognos* OR predict* OR clinical* OR outcome* OR associa* OR risk* OR death OR mortal* OR surviv* OR follow-up OR course OR progress* OR deteriorat*) | 1371620 |
| #11 | MeSH descriptor: [Prognosis] explode all trees | 148085 |
| #12 | MeSH descriptor: [Mortality] explode all trees | 13004 |
| #13 | MeSH descriptor: [Survival] explode all trees | 130 |
| #14 | #10 OR #11 OR #12 OR #13 | 1371771 |
| #15 | #5 AND #9 AND #14 | 90 |

#

# Appendix 2: Data collection and analysis

### Selection of studies

One author (SA) screened titles and abstracts and retrieved the full texts of all potentially eligible studies. The full texts were reviewed and studies meeting the inclusion criteria were included after discussion with another author (AJS). The selection process was recorded in a Preferred Reporting Items for Systematic Reviews and Meta-Analyses (PRISMA) flow diagram.

### Data extraction and management

Data extraction and risk of bias analysis were performed independently by two review authors (SA and FAA), and disagreements were discussed with (AJS). Data were extracted and collated data using a standardised extraction form. The methodological quality of the included studies was assessed using a modified Quality In Prognosis Studies tool (QUIPS) (1). The corresponding authors of all included studies in the meta-analysis were contacted for hazard ratios (HRs) of unpublished CMR measurements. The unadjusted HRs were thought when bivariate, multivariate or adjusted % predicted HRs were reported. For the meta-analysis, steps were taken to present data only for patients with PAH; where clarification was required, study authors were contacted directly.

### Statistical analysis and data synthesis

HRs with 95% confidence intervals (CIs) of unadjusted univariate event-free survival regression analyses for CMR measurements including right and left ventricular ejection fraction (RVEF and LVEF), RV and LV mass index (RVMI and LVMI), RV and LV end-diastolic volume index (RVEDVI and LVEDVI), RV and LV end-systolic volume index (RVESVI and LVESVI) and RV and LV stroke volume index (RV SVI and LV SVI), were pooled. Published and unpublished data were included in all meta-analyses. Meta-analyses of HRs were conducted using Review Manager 5.4 (The Cochrane Collaboration, 2020) using a random‐effect model with 95% CI. Forest plots of the baseline CMR measurements were presented using GraphPad Prism version 8.3 (GraphPad Software, La Jolla CA, USA). Participant characteristics were presented as mean ± standard deviation (SD). If the median and ranges were reported for demographics and baseline CMR metrics, data were expressed as mean ± SD, using standard approaches (2). Means and SDs were pooled using the formula provided in Table 7.7.a in the Cochrane Handbook (3). Between-study heterogeneity was measured using the I² statistic. An I^2^ > 50 was considered as high, and an I^2^ > 30 as moderate heterogeneity. Meta-regression analyses were performed to investigate age, gender, 6-minute walking test and RHC parameters as study‐level covariates on CMR measurements that had a moderate or high statistical heterogeneity. Meta-regression was performed using SPSS Statistics 26, (IBM Corp., Armonk, N.Y., USA). Publication bias was assessed graphically using funnel plots where at least ten studies were included in a meta-analysis.

#

# Appendix 3: Results of the literature search

The systematic literature search identified 10,939 citations. Deduplication left 8,119 citations; the majority of studies were excluded from the title and abstract screening because they i) did not meet the inclusion criteria, primarily due to the absence of prognostic data, ii) did not include patients with pulmonary arterial hypertension (PAH) or iii) were based on echocardiographic or right heart catheter (RHC) metrics and did not report magnetic resonance imaging (MRI) metrics. The full texts of 105 articles were retrieved for more detailed evaluation, of which a further 83 were excluded because they were conference abstracts, MRI findings were not described, included children, pulmonary hypertension (PH) other than PAH, did not perform univariate Cox regression analysis or studies with cohorts included in later publications by the same author group. The PRISMA flow diagram of the literature search is shown in supplementary Figure 1.


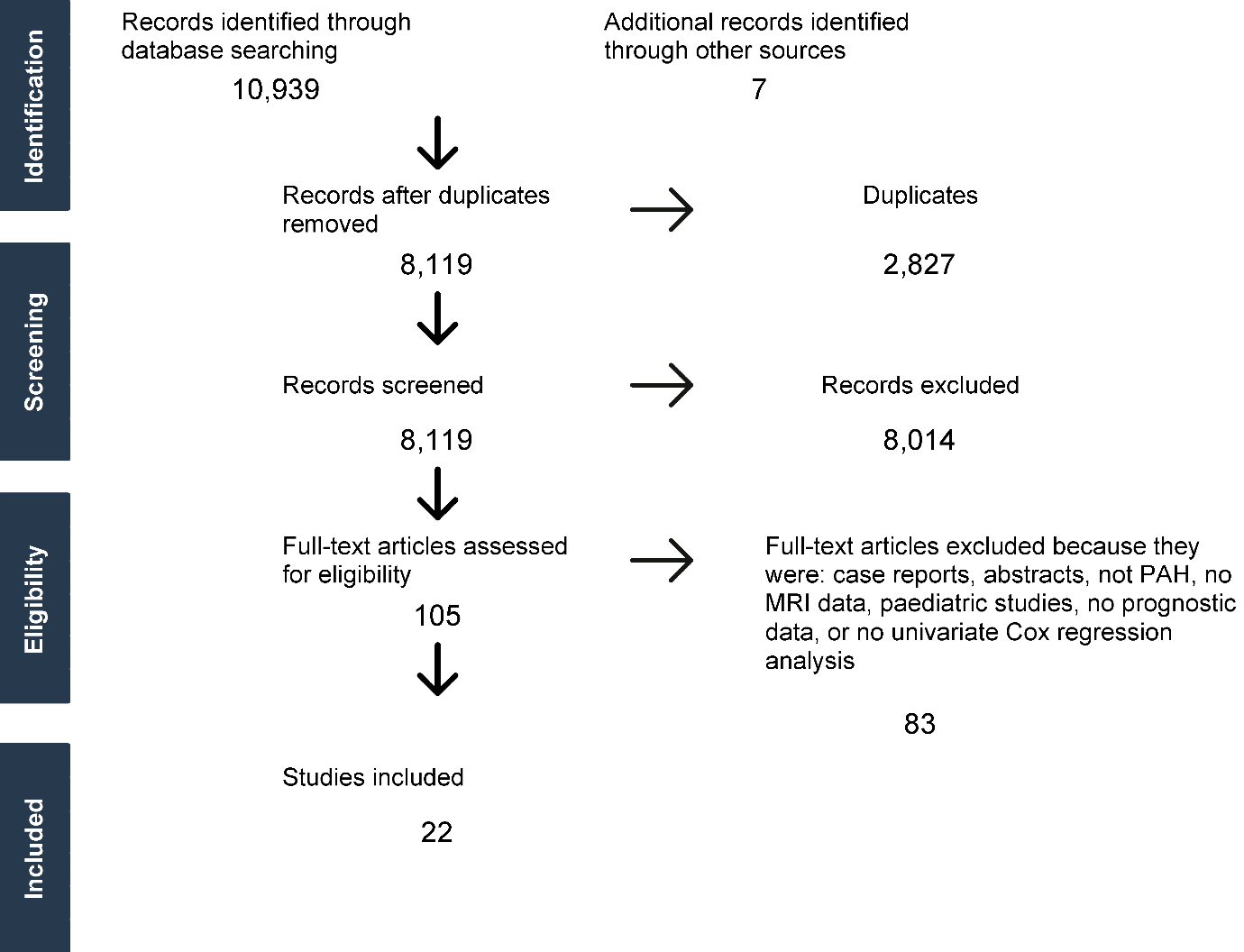


Figure 1 PRISMA flow diagram of the literature search.

# Appendix 3: Quality In Prognosis Studies (QUIPS) risk of bias assessment

The risk of bias per study is summarised in the risk of bias figure (supplementary Figure 2).


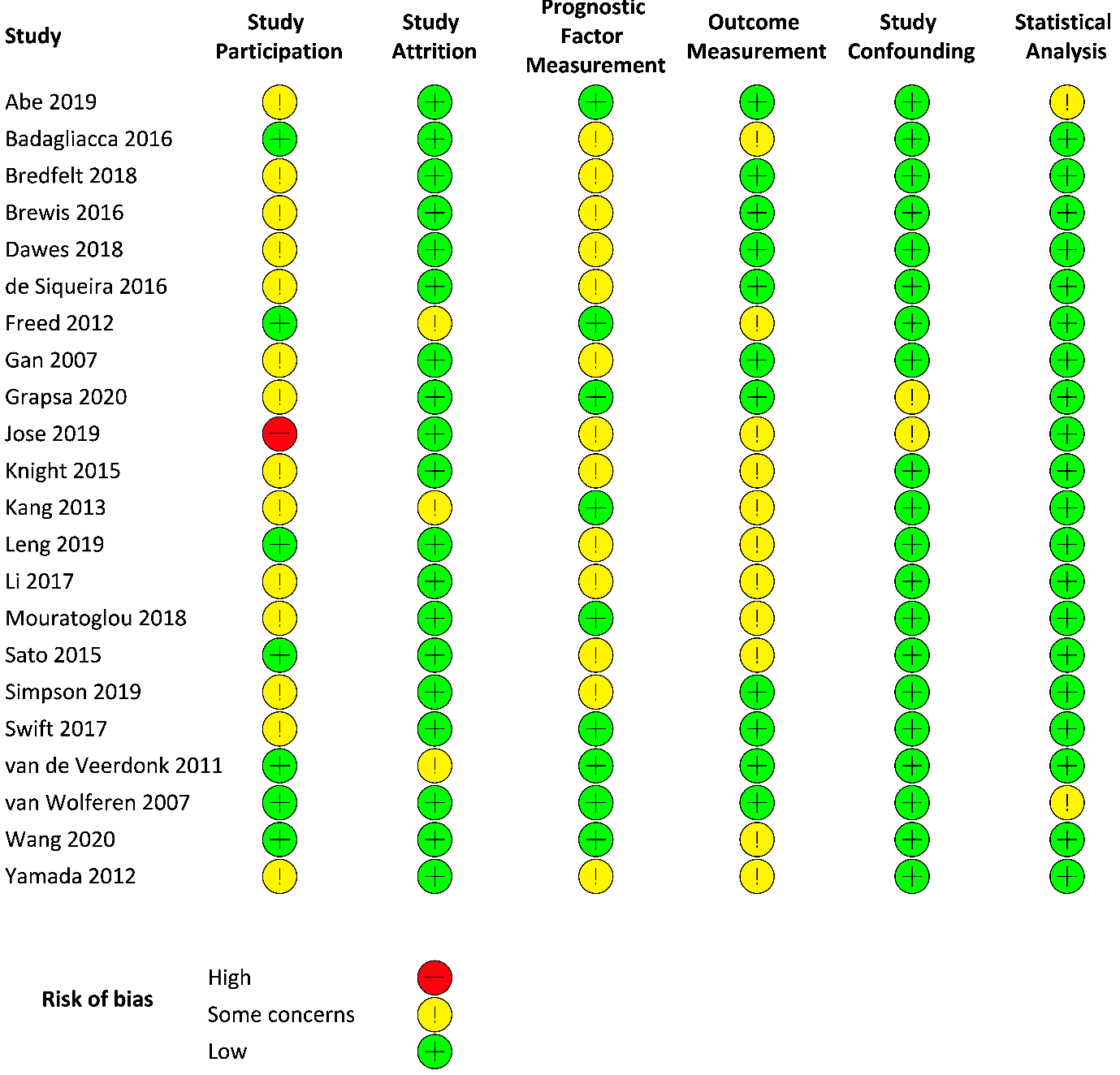


Figure 2 Risk of bias summary for each included study. Jose 2019 was rated to have a high risk of bias in the participation domain due to the small number of patients (18) with PAH.

### Study participation

Seven studies were rated as a low risk of bias as they had a prospective design and recruited more than 50 consecutive patients. The remainder of the studies were assessed to have some concerns as they were retrospective studies or had a small study size of fewer than 50 patients. In retrospective studies, it is not possible to control the way data is collected and may include missing data, confounding and selective outcome reporting (4). Jose 2019 was rated to have a high risk of bias as less than 20 participants had pulmonary arterial hypertension (PAH) (5).

### Study attrition

Twenty-two studies reported no loss to follow-up. Seven studies reported a loss of follow-up and explained the reason for it. Loss of follow-up of more than 10% was judged to represent some concerns for bias (Freed 2012 (6); Kang 2013 (7); van de Veerdonk 2011 (8)). No study had more than 20% attrition which was judged as a high risk of bias.

### Prognostic factor measurement

Nine studies were judged to have a low risk of bias as they reported blinding of the cardiac MRI (CMR) assessor to clinical and outcome data. In these studies, intra-observer and interobserver variability were also analysed by having a random sample of CMRs read by a second CMR assessor. Thirteen studies did not report blinding of the CMR assessor and were judged to have some concerns for bias.

### Outcome measurement

Twelve studies were judged to have a low risk of bias for outcome measurement if they either reported mortality only as the study end-point or reported blinding of the outcome assessor if they included other clinical outcomes in addition to death. Ten studies were rated to have some concern for bias as they included clinical worsening outcomes in addition but not blind the outcome assessor to patient data.

### Study confounding

All included studies reported important patient characteristics and clinical measurements including age, sex, disease subtype, RHC findings and 6-min walk distance (6MWD). Nineteen studies also assessed the effects of confounding factors on the prognostic factors using multivariate regression analysis and were rated as having a low risk of bias. One study did not perform a multivariate analysis and was rated to have some concerns for confounding bias.

### Statistical analysis

The statistical methods were adequately explained in all studies. In the studies included in the meta-analysis, Abe 2019 (9) was rated as having some concerns for bias as they excluded patients from the univariate and multivariate analysis if they did not have a clinical event. All studies included in the meta-analysis presented univariate Cox proportional hazards ratios (HR) for CMR measurements. The van Wolferen 2007 (12) had extremely wide confidence intervals and very large effect sizes which are at odds with the remainder of the included studies. The discrepancy is probably due to van Wolferen 2007 reporting CMR measurements scaled to the change per standard deviation rather than per unit change.

# Appendix 4: Meta-regression for possible sources of heterogeneity

A series of meta-regression analyses were performed to explore the increase in logHR per unit increase in the covariate, weighted by the inverse variance of the HRs. CMR measurements with moderate and high statistical heterogeneity were RVEF, RVEDVI, RVSVI, LVEDVI, LVESVI and LVMI. The meta-regression coefficients were not statistically significant for age, male gender, 6MWD or RHC (cardiac index, mean right atrial pressure and pulmonary vascular resistance) in any of the analyses performed suggesting no linear relationship between the prognostic effect of cardiac measurements and these covariables (Table 1). There was not enough data to perform a meta-regression for functional class, disease stage or treatment status.

Table 1 Meta-regression of age and proportion of men (%) on logarithmic hazard ratio for CMR measurements with moderate to high statistical heterogeneity

| **CMR measurement** | **Covariate coefficient (P value)** | | | | | |
| --- | --- | --- | --- | --- | --- | --- |
|  | **Age** | **Male gender** | **6MWD** | **CI** | **mRAP** | **PVR** |
| RVEF | 0.40 (0.08) | -0.11 (0.64) | -0.45 (0.10) | 0.13 (0.64) | 0.44 (0.09) | 0.52 (0.10) |
| RVEDVI | -0.04 (0.88) | -0.18 (0.46) | 0.01 (0.98) | -0.17 (0.56) | -0.29 (0.29) | -0.42 (0.20) |
| RVSVI | -0.02 (0.95) | 0.23 (0.44) | insufficient data | 0.39 (0.27) | 0.29 (0.41) | insufficient data |
| LVEDVI | -0.24 (0.40) | -0.13 (0.63) | 0.06 (0.86) | 0.07 (0.82) | -0.25 (0.43) | insufficient data |
| LVESVI | -0.206 (0.50) | -0.32 (0.26) | -0.39 (0.27) | -0.20 (0.58) | -0.41 (0.24) | insufficient data |
| LVMI | 0.28 (0.39) | -0.49 (0.11) | insufficient data | 0.35 (0.36) | 0.27 (0.48) | insufficient data |

6MWD, 6-minute walking distance; mRAP, mean right atrial pressure; CI, cardiac index; PVR, pulmonary vascular resistance (Wood units)

# Appendix 5: Unpublished data

Most studies included in the meta-analysis were contacted for unpublished data. The study authors of 13 included studies kindly replied to our requests for additional data where they published results for a mixed PH cohort (Dawes 2018 (10), de Siqueira 2016 (13), Jose 2019 (5)), bivariate or multivariate hazard ratios (HRs) (Brewis 2016 (14)), adjusted % predict HRs (Swift 2017 (15)), non-indexed volumetric measurements (Badagliacca 2016 (16)) or reported a subset of CMR indices (Abe 2019 (9), Bredfelt 2018 (17), Knight 2015 (11), Simpson 2019 (18), Leng 2019 (19), Mouratoglou 2018 (20), Van de Veerdonk 2011 (8)). The authors provided us with PAH results only, univariate, unscaled and non-adjusted HRs, indexed volumetric measurements and the results for additional CMR metrics. Five studies published prior to 2015 reporting only a few CMR metrics did not respond to our requests for additional information. The results of Van Wolferen 2007 (12) had very large effect sizes and standard errors, following discussion with the co-authors we understand this is due to scaling of the CMR measurements to the standard deviation rather than the unit of measurement. We therefore decided not to pool the results of Van Wolferen 2007 with the rest of the studies due to the different unit of scaling used.

The unpublished univariate Cox regression HRs of these studies are provided in Table 2. The meta-analyses results using only published data are shown in Table 3. The published data only results show slightly larger effect sizes and are less precise with wider confidence intervals.

Table 2 Unpublished univariate hazard ratios provided by study authors


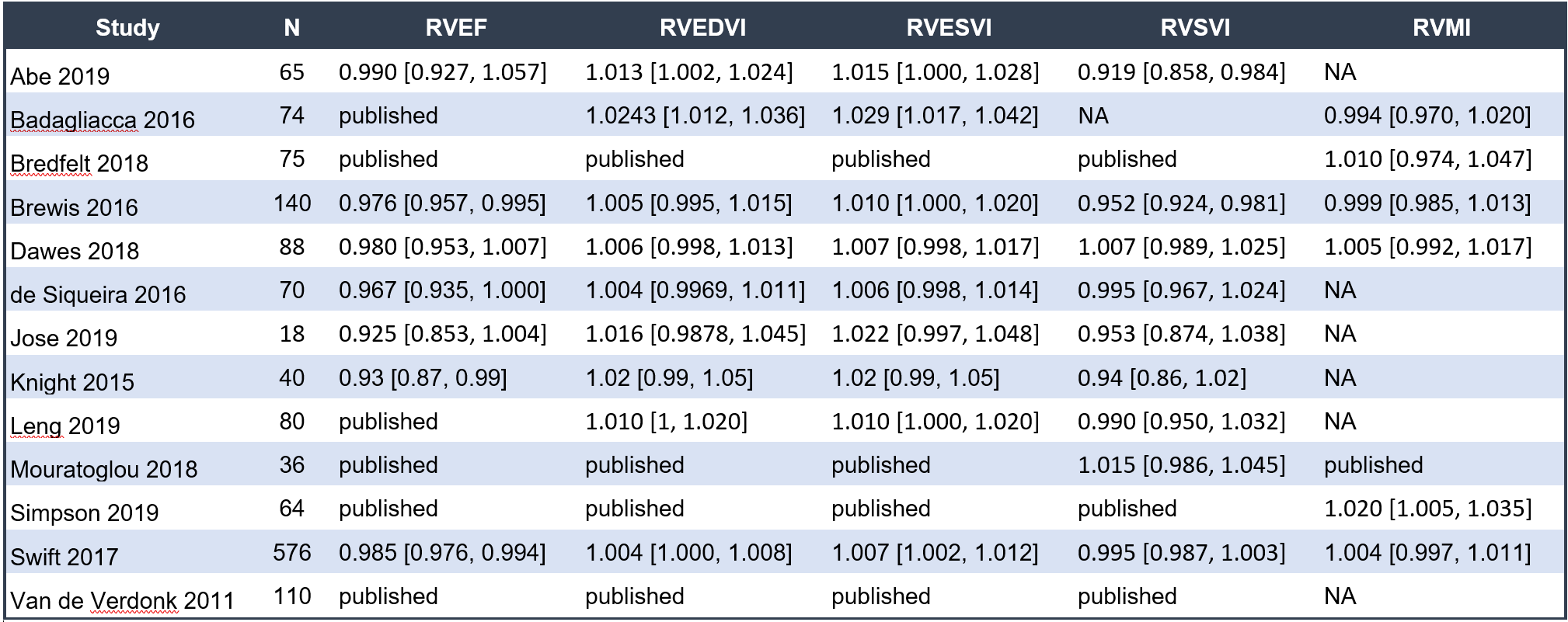

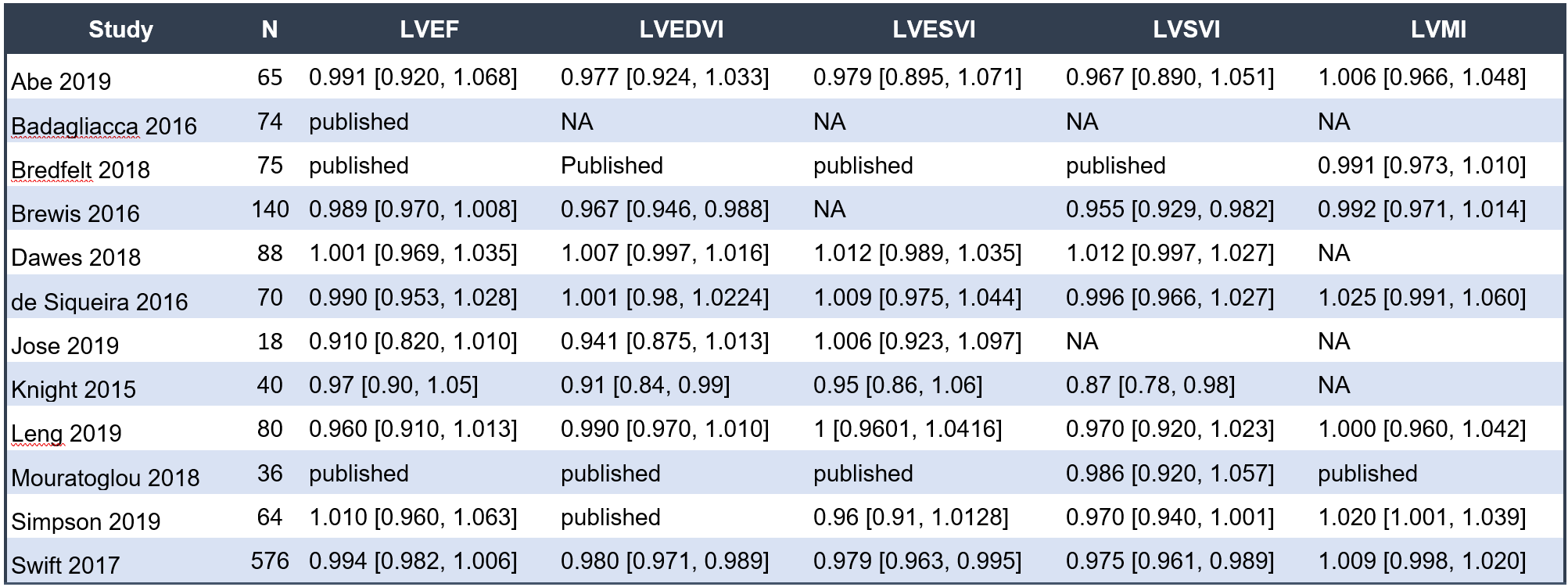


Table 3 Meta-analysis results of published data only.

# Appendix 6: Funnel Plots

Publication bias was assessed graphically using funnel plots where at least ten studies were included in a meta-analysis (suppl Figure 3). The funnel plots for RVEF, LVEF and LVEDVI and RV mass index showed some minor asymmetry which might that some small studies with extreme effects were not published.


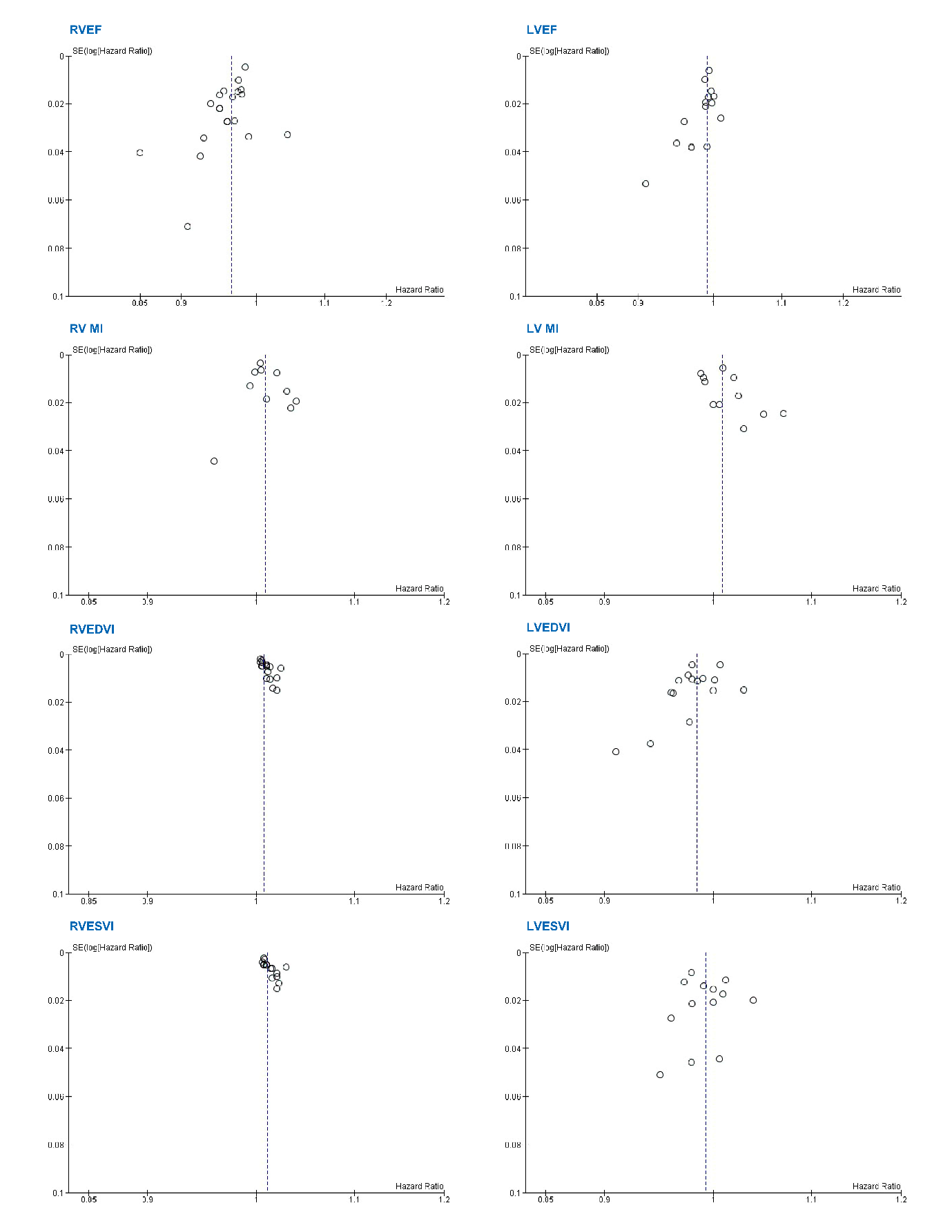


Figure 3 Funnel plots of the meta-analyses of CMR measurements.
Minor asymmetry is seen in the funnel plots of RVEF, LVEF, RVMI and LVEDVI which may indicate that a small study with extreme effects was not published.

# Appendix 7: Additional Meta-Analyses

### **Right atrial measurements**

Right atrial (RA) volume is not a significant prognostic marker (HR 1.01, 95% CI 1.00 to 1.02; p = 0.09; participants = 201; studies = 3). RA area index is a significant prognostic marker (HR 1.08, 95% CI 1.04 to 1.12; p = 0.0001; participants = 106; studies = 2).

Although the European Society of Cardiologists and European Respiratory Society guidelines include RA size and the presence of pericardial effusion on echocardiogram as prognostic factors in PAH, there is only limited data on these metrics as a prognostic marker using CMR (21). RA volume on CMR has been evaluated in two studies only. The meta-analysis of their results did not reach statistical significance; however, the direction of effect suggests that an increased RA volume is associated with a poor outcome. Further studies assessing CMR RA volume and its relation to clinical worsening and prognosis in PAH prognosis are needed. Two additional RA CMR markers that have been reported to be prognostically significant but could not be studied in the meta-analysis as only one study reported each metric; these were RA emptying fraction and RA strain (19,22). RA emptying fraction is the difference in the RA _max_ - RA _min_ volume divided by RA _max_ volume and RA strain was calculated as the distance between the posterior RA wall and the atrioventricular junction on the 4-chamber view.

### **Pulmonary artery measurements**

Surrogate markers for pulmonary artery (PA) stiffness such as relative area change (RAC) and PA distensibility were reported in two studies only (15,23). The meta-analysis showed that PA distensibility was prognostically significant but PA RAC did not reach statistical significance (p=0.05). Although PA RAC was significant in each study individually, their pooled result was imprecise and had wide confidence intervals.The PA RAC meta-analysis result was HR 0.92, 95% CI 0.85 to 1.00; p = 0.05; participants = 646; studies = 2).

### **Stroke volume / RV end-systolic volume**

In CTD-PAH, PA RAC and load-independent measurements such as RV-PA coupling measurements were significantly prognostic (9,15,18,24). Four studies, including 845 participants, reported the stroke volume divided by RV end-systolic volume to estimate RV elastance (Ees) divided by PA elastance (Ea). The pooled CMR Ees / Ea ratio is a significant prognostic marker (HR 0.47, 95% CI 0.33 to 0.68; p < 0.00001).

# References

1. Hayden JA., Côté P., Bombardier C. Evaluation of the Quality of Prognosis Studies in Systematic Reviews. Annals of Internal Medicine 2006:427. Doi: [10.7326/0003-4819-144-6-200603210-00010](http://dx.doi.org/10.7326/0003-4819-144-6-200603210-00010).

2. Wan X., Wang W., Liu J., Tong T. Estimating the sample mean and standard deviation from the sample size, median, range and/or interquartile range. BMC Med Res Methodol 2014;14(135):135.

3. Higgins JPT., Deeks JJ. Selecting Studies and Collecting Data. Cochrane Handbook for Systematic Reviews of Interventions n.d.:151–85. Doi: [10.1002/9780470712184.ch7](http://dx.doi.org/10.1002/9780470712184.ch7).

4. Sica GT. Bias in research studies. Radiology 2006;238(3).

5. Jose A., Kher A., O’Donnell RE., Elwing JM. Cardiac magnetic resonance imaging as a prognostic biomarker in treatment-naïve pulmonary hypertension. Eur J Radiol 2020;123:108784.

6. Freed BH., Gomberg-Maitland M., Chandra S., et al. Late gadolinium enhancement cardiovascular magnetic resonance predicts clinical worsening in patients with pulmonary hypertension. J Cardiovasc Magn Reson 2012;14(11):11.

7. Kang K-W., Chang H-J., Yoo YP., et al. Cardiac magnetic resonance-derived right ventricular outflow tract systolic flow acceleration: a novel index of right ventricular function and prognosis in patients with pulmonary arterial hypertension. Int J Cardiovasc Imaging 2013;29(8):1759–67.

8. van de Veerdonk MC., Kind T., Marcus JT., et al. Progressive right ventricular dysfunction in patients with pulmonary arterial hypertension responding to therapy. J Am Coll Cardiol 2011;58(24):2511–9.

9. Abe N., Kato M., Kono M., et al. Right ventricular dimension index by cardiac magnetic resonance for prognostication in connective tissue diseases and pulmonary hypertension. Rheumatology 2020;59(3):622–33.

10. Dawes TJW., Cai J., Quinlan M., et al. Fractal Analysis of Right Ventricular Trabeculae in Pulmonary Hypertension. Radiology 2018;288(2):386–95.

11. Knight DS., Steeden JA., Moledina S., Jones A., Coghlan JG., Muthurangu V. Left ventricular diastolic dysfunction in pulmonary hypertension predicts functional capacity and clinical worsening: a tissue phase mapping study. J Cardiovasc Magn Reson 2015;17(116):116.

12. van Wolferen SA., Marcus JT., Boonstra A., et al. Prognostic value of right ventricular mass, volume, and function in idiopathic pulmonary arterial hypertension. Eur Heart J 2007;28(10):1250–7.

13. de Siqueira MEM., Pozo E., Fernandes VR., et al. Characterization and clinical significance of right ventricular mechanics in pulmonary hypertension evaluated with cardiovascular magnetic resonance feature tracking. J Cardiovasc Magn Reson 2016;18(1):39.

14. Brewis MJ., Bellofiore A., Vanderpool RR., et al. Imaging right ventricular function to predict outcome in pulmonary arterial hypertension. Int J Cardiol 2016;218:206–11.

15. Swift AJ., Capener D., Johns C., et al. Magnetic Resonance Imaging in the Prognostic Evaluation of Patients with Pulmonary Arterial Hypertension. Am J Respir Crit Care Med 2017;196(2):228–39.

16. Badagliacca R., Poscia R., Pezzuto B., et al. Right ventricular concentric hypertrophy and clinical worsening in idiopathic pulmonary arterial hypertension. J Heart Lung Transplant 2016;35(11):1321–9.

17. Bredfelt A., Rådegran G., Hesselstrand R., Arheden H., Ostenfeld E. Increased right atrial volume measured with cardiac magnetic resonance is associated with worse clinical outcome in patients with pre-capillary pulmonary hypertension. ESC Heart Fail 2018;5(5):864–75.

18. Simpson CE., Damico RL., Kolb TM., et al. Ventricular mass as a prognostic imaging biomarker in incident pulmonary arterial hypertension. Eur Respir J 2019;53(4). Doi: [10.1183/13993003.02067-2018](http://dx.doi.org/10.1183/13993003.02067-2018).

19. Leng S., Dong Y., Wu Y., et al. Impaired Cardiovascular Magnetic Resonance-Derived Rapid Semiautomated Right Atrial Longitudinal Strain Is Associated With Decompensated Hemodynamics in Pulmonary Arterial Hypertension. Circ Cardiovasc Imaging 2019;12(5):e008582.

20. Mouratoglou SA., Kallifatidis A., Pitsiou G., et al. Duration of interventricular septal shift toward the left ventricle is associated with poor clinical outcome in precapillary pulmonary hypertension: A cardiac magnetic resonance study. Hellenic J Cardiol 2018. Doi: [10.1016/j.hjc.2018.10.007](http://dx.doi.org/10.1016/j.hjc.2018.10.007).

21. Galiè N., Humbert M., Vachiery J-L., et al. 2015 ESC/ERS Guidelines for the diagnosis and treatment of pulmonary hypertension: The Joint Task Force for the Diagnosis and Treatment of Pulmonary Hypertension of the European Society of Cardiology (ESC) and the European Respiratory Society (ERS): Endorsed by: Association for European Paediatric and Congenital Cardiology (AEPC), International Society for Heart and Lung Transplantation (ISHLT). Eur Respir J 2015;46(4):903–75.

22. Darsaklis K., Dickson ME., Cornwell W 3rd., et al. Right atrial emptying fraction non-invasively predicts mortality in pulmonary hypertension. Int J Cardiovasc Imaging 2016;32(7):1121–30.

23. Gan CT-J., Lankhaar J-W., Westerhof N., et al. Noninvasively assessed pulmonary artery stiffness predicts mortality in pulmonary arterial hypertension. Chest 2007;132(6):1906–12.

24. Hagger D., Condliffe R., Woodhouse N., et al. Ventricular mass index correlates with pulmonary artery pressure and predicts survival in suspected systemic sclerosis-associated pulmonary arterial hypertension. Rheumatology 2009;48(9):1137–42.
